# Special Issue “Flow Cytometry: Applications and Challenges”

**DOI:** 10.3390/ijms27177899

**Published:** 2026-09-04

**Authors:** Alessandra Falda

**Affiliations:** Laboratory Medicine Unit, Integrated Diagnostic Services-DIDAS, Padua University Hospital, 35128 Padua, Italy; alessandra.falda@aopd.veneto.it

Despite the use of molecular tests and imaging techniques, flow cytometry continues to capture attention in various fields today [1,2].

Artificial intelligence and precision medicine are pushing medical knowledge to its limits, and within this integrated landscape, flow cytometry remains a key contributor. With proteomics and sequencing, high-sensitivity multiparametric analysis targets the immune system and signaling pathways (contribution 4) [3] and enables minimal residual disease (MRD) assessments to guide treatment (contribution 3) [4]; the study of microenvironments and extracellular vesicles via expanded panels [5,6] illuminates immune modulation and new therapeutic targets [7,8,9].

The contributions gathered in this Special Issue share a clear underlying thread: the ability of flow cytometry to reveal innovative and informative cellular features across a wide range of biological processes.

The twelve works in this collection can be grouped according to four thematic lenses (Figure 1): (i) the diagnosis and monitoring of hematological malignancies; (ii) technological and methodological innovation; (iii) immune cell biology and functional phenotyping; and (iv) the expansion of flow cytometry into new applied domains.

By theme: hematological diagnosis—fluorescent aerolysin (FLAER) (contribution 1), T-cell receptor β-chain constant region (TRBC) 1 clustering (contribution 2), and MRD antigen panels (contribution 3); methodological innovation—spectral cytometry (contribution 4), single-organelle mitochondrial analysis (contribution 5), quantitative assays (contribution 6), and FlowCLOc (contribution 7); functional immune phenotyping—hematopoietic stem and progenitor cell (HSPC) protocols (contribution 8), plasmacytoid dendritic cell (pDC) identification (contribution 9), and macrophage plasticity (contribution 10); and new applied domains—the basophil activation test for wheat allergy (contribution 11) and grape must fermentation monitoring (contribution 12).

Álvarez Flores et al. (contribution 1) posit that the inclusion of FLAER reveals reduced or absent binding in the clonal precursors of acute leukemias, most markedly in acute promyelocytic leukemia (APL): across 71 malignancies, APL promyelocytes had significantly lower FLAER expression than other myeloid precursors. FLAER also tracked MRD, revealing FLAER-negative populations preceding relapse. As it detects glycosylphosphatidylinositol (GPI) anchors (lost in paroxysmal nocturnal hemoglobinuria, PNH), reduced binding may reflect altered GPI biosynthesis or immune evasion, and the use of PML-RARA refines APL diagnoses.

Complementing this, Karela et al. [10] demonstrate that normal marrow physiologically harbors immature myeloid precursors (CD34−/CD117+/HLA-DR+/CD33+) with reduced (FLAER-dim) intensity, whereas true FLAER-negative populations remain confined to the maturing compartments of PNH. This reproducible, maturation-dependent pattern could serve as a marker of hematopoietic hierarchy in diagnostic and MRD monitoring protocols.

This builds on an earlier warning by Sutherland et al. [11]: reduced FLAER reactivity in blasts should not be read as evidence of PNH, since in some acute leukemias it reflects an aberrant myeloid phenotype with normal granulocytes—pointing to clonal myeloid disease rather than GPI-anchor deficiency.

An additional theme is T-cell clonality assessed via TRBC1/TRBC2. Buček et al. (contribution 2) reveal that unsupervised clustering (Phenograph, t-SNE) of TRBC1 flow cytometry outperforms manual gating: across 275 samples this approach identified monotypic T-cell populations in 72.4% of cases versus 65.1% by manual gating and 68.0% by PCR, detecting clones missed by both techniques.

Extending this across specimen types, Berg et al. [12] evaluated TRBC1 expression in diverse tissues and body fluids, confirming TRBC1 flow cytometry as a robust, specific tool for T-cell clonality assessment across clinical settings.

The rationale is well defined: Chaudhary et al. [13] demonstrate that polyclonal α/β T-cells display mixed TRBC1/TRBC2 expression, whereas clonal expansions are restricted to a single isoform. Building on this, Soilleux et al. [14] validate anti-TRBC1/2 immunohistochemistry as a rapid, cost-effective surrogate for PCR-based clonality testing, documenting T-cell monotypia directly in formalin-fixed, paraffin-embedded (FFPE) sections while preserving the morphologic context.

Returning to flow cytometry, Horna et al. [15] prove that dual TRBC1/TRBC2 staining enables rapid single-tube assays—analogous to κ/λ evaluation in B cells—removing TRBC1-dim artifacts and identifying the targetable isoform for routine diagnostics.

Taylor et al. [16] observe that TRBC1/TRBC2 immunophenotyping achieves optimal sensitivity for T-cell malignancies when applied to well-defined or phenotypically aberrant subsets, underscoring the role of accurate gating. This approach provides information that is complementary to conventional markers and molecular clonality assays, strengthening the diagnostic framework without replacing established methods.

Semchenkova et al. (contribution 3) establish a practical multicolor panel for MRD monitoring in T-cell acute lymphoblastic leukemia (T-ALL). Across 198 pediatric patients, CD3, CD4, CD5, CD7, CD8, and CD45 were strongly therapy-modulated, whereas CD48 and CD99 stayed stable and reliable for late MRD—yielding a near-universal panel that minimizes customization and improves sensitivity and specificity compared to earlier algorithms.

Complementing this, the 12-color, 2-tube Next-Generation Flow panel of the EuroFlow consortium [4] delivers standardized, high-sensitivity (≤10^−5^) MRD detection across virtually all T-ALL subtypes, including ETP-ALL. Validated in 96 patients across seven centers, it exhibited excellent inter-tube concordance (R^2^ = 0.99) and a 97.5% agreement with allele-specific oligonucleotide quantitative PCR, supporting its integration into routine clinical protocols.

Extending clonality assessment to immature, CD3-negative proliferations, Xi et al. [17] demonstrate that cytoplasmic TRBC1 flow cytometry reliably evaluates clonality in T-ALL and mature T-cell lymphomas, such as angioimmunoblastic T-cell lymphoma: neoplastic lymphoblasts exhibit restricted (monotypic) cyTRBC1 versus the polytypic pattern of normal thymocytes, expanding TRBC1 analysis beyond surface staining.

At the methodological core, Piasecki et al. (contribution 5) extend fluorescence-activated mitochondria sorting into a versatile nanoscale flow cytometry platform for single-organelle analysis. Their assays identify mitochondrial subpopulations, track PINK1/Parkin translocation as a readout of membrane potential and mitophagy, quantify mitochondrial DNA at single-organelle resolutions, and characterize both free-floating mitochondria and those within extracellular vesicles—capabilities largely inaccessible to conventional methods.

Two studies demonstrate organelle-level readouts in disease: Liu et al. [18] find that impaired PINK1/Parkin mitophagy drives mitochondrial damage and stromal invasiveness in adenomyosis, reversed by Guizhi Fuling Wan; Mahata et al. [19] reveal that a carvacrol-loaded polydopamine–TPGS–transferrin nanoconjugate triggers oxidative and apoptotic responses in MCF-7 cells.

Xiao et al. [20] bridge these perspectives with MitoATP-nFCM, a nano-flow cytometry assay that quantifies ATP in individual mitochondria. Isolated tumor mitochondria exhibit markedly elevated mitoATP (1.7–1.9-fold), raised Δψm, upregulated ATP synthase, and reduced HK2—an OXPHOS-dependent phenotype that challenges the Warburg paradigm—while enabling high-precision screening of selective mitochondrial inhibitors free of cytosolic interference. With Piasecki’s work, this establishes mitochondria as heterogeneous populations demanding organelle-resolved analysis.

Spectral flow cytometry is another theme: Astakhova et al. (contribution 4) examine how acquiring the full emission spectrum with spectral unmixing expands measurable markers, simplifies panel design, and reduces overlap artifacts, while considering unmixing accuracy, standardization, and data handling constraints.

Building on this, Olofsson and Karlsson [3] offer a practical guide to studying antigen-specific T-cell responses, where spectral resolution is decisive for rare populations, detailing the choices that determine data quality—stimulation strategy, autofluorescence control, titration, and reference controls—and pairing tailored gating with computational tools (t-SNE, UMAP, FlowSOM, SPICE).

Seo et al. [21] translate this into a 25-parameter panel that resolves innate lymphoid cells (ILCs) in murine mammary tumors. Distributing lineage markers across spectrally distinct fluorochromes discriminates NK cells and ILC1–3 alongside T and B lymphocytes; in the PyMT model, tumor-associated ILC2s exhibit higher PD-1 and Ki67, indicating functional modulation. A reproducible pipeline and validation across Cytek Aurora and Sony ID7000 confirm that the workflow is robust and implementable.

Robinson et al. [1], pioneers of spectral cytometry, frame this tool as the natural evolution of polychromatic analysis: full-spectrum capture with over-determined unmixing supports 40–50-fluorochrome panels; reduces spreading errors; and increases detectable features to include enzymatic, nucleic acid, and structural readouts. They emphasize panel quality metrics and the unresolved challenge of cross-platform portability, so rigorous empirical optimization remains essential even with automated design tools.

Möllenkamp et al. [22] demonstrate the clinical payoff in multiple sclerosis, where high-parameter profiling identifies immune endophenotypes; distinguishes compartments, with cerebrospinal fluid carrying stronger specific inflammatory signals than blood; and supports prognostic stratification, the prediction of anti-CD20 responses, and treatment monitoring.

Liu et al. [2] reaffirm spectral cytometry as transformative—using panels with more than forty parameters with dedicated autofluorescence correction—while highlighting calibration and harmonized pipelines, and Isbell et al. [5] make it accessible through a guided 23-parameter Cytek Aurora panel with shared reagents, templates, and training protocols.

The protocol developed by Schmachtel et al. (contribution 8) delivers a practical roadmap for isolating human HSPCs via fluorescence-activated cell sorting (FACS). Addressing the rarity of long-term repopulating HSCs, it defines authentic LT-HSCs as lin^−^CD34^+^CD38^−^CD45RA^−^CD90^+^CD49f^+^ and specifies hierarchical gating, marker combinations, essential controls, and careful pre-analytics for reliable enrichment from mobilized peripheral blood.

Girondier et al. [23] expand this with a 19-marker spectral panel that resolves, in a single murine bone marrow sample, HSCs, myeloid and lymphoid progenitors, all erythroid maturation stages, and mature populations. Built on quantitative criteria (similarity index < 0.85, autofluorescence characterization) and read with UMAP, it captures continuous differentiation trajectories. Validation with pathological models (aged animals; β-thalassemia) and channels reserved for GFP/mCherry make it well suited for preclinical gene therapy readouts.

The two works are complementary: Schmachtel’s protocol sets a standard for purifying authentic human HSCs, whereas Girondier’s spectral approach extends analysis to the whole hematopoietic ecosystem. Echoing the 17-color workflow of Kiel et al. [6], they converge on one message: robust cytometry depends on informed technical choices; systematic titration; appropriate controls; and reproducible, transferable gating criteria.

A complementary study in *Nature Biotechnology* [24] ensures that only correctly integrated cells are retained after homology-directed repair: the SMArT platform pairs a transient tTA-activated reporter (SMArT-1) with a marker linked to the corrective cDNA and amplified by a TALE-VPR transactivator (SMArT-2), validated in thymic organoids and NBSGW mice to yield fully HDR-edited grafts.

These efforts are complementary: Schmachtel’s protocol established how to obtain phenotypically pure HSPCs, while SMArT indicates what edited cells to keep—optimizing cellular input and genetic output, respectively. Together they sketch an ideal gene-therapy pipeline—LT-HSC purification, HDR editing, SMArT-based selection, and functional validation—that could improve genomic safety and preserve physiological regulation of corrected genes such as IL2RG or CD40LG, marking a decisive step toward safer cellular products.

Reliable identification is a distinct challenge: Demeter et al. (contribution 9) demonstrate that prolonged TLR7/9 stimulation induces BDCA-4 (CD304) on non-pDCs (chiefly CD14^+^ monocytes), eroding single-marker specificity, and validate a refined strategy coupling BDCA-4 with a small exclusion marker cocktail for reliable pDC discrimination in two channels.

Lemke-Miltner et al. [25] contribute a therapeutic dimension, showing that BDCA2 is not merely a marker but an operative receptor governing pDC engagement with vidutolimod, a TLR9 agonist nanoparticle for cancer immunotherapy. The degree of anti-Qβ opsonization balances BDCA2 engagement with robust pDC activation and internalization, with the suppression of the type I interferon response—an insight from imaging flow cytometry that informs pDC-targeting nanoparticle design.

Extending beyond pDCs, Yang et al. [26] demonstrate that conventional dendritic cells exhibit unexpected plasticity: in mice, hybrid and macrophage-like subsets blur canonical cDC taxonomy, confirming that low-parameter phenotyping cannot capture true myeloid heterogeneity. A common lesson emerges: stimulus-induced receptor modulation couples cellular function to detectability, so the identification and biological interpretation of dendritic cells cannot be considered independently.

Finally, Liu et al. [27] document the limited whole-blood stability of dendritic cell markers and the resulting misclassification risk and verify stability, precision, linearity, and the reportable range—with reference intervals for dendritic cells, γδ T-cells, and follicular helper T-cells—advancing multiparametric DC panels toward routine laboratory work.

Sizzano et al. (contribution 12) bring flow cytometry to the winery, monitoring microbial dynamics during Pinot Noir must fermentation under three inoculation strategies. Cytometry reliably tracks yeast viability and detects viable bacteria throughout fermentation, delivering near-real-time readouts for process control corroborated by amplicon sequencing—a practical, high-throughput complement to molecular surveys in enology.

Conacher et al. [28] approach the same ecosystem mechanistically, developing a multicolor platform in which stable genomic fluorescent tags (GFP, BFP, RFP) with hierarchical gating discriminate individual strains within synthetic yeast consortia. Coupled with sorting, it resolves growth trajectories, physical interactions, and competition at the species level while reducing the biases of plating or ARISA—providing a robust experimental tool for assessing yeast ecology under defined conditions.

Onetto et al. [29] explain the early dominance of non-Saccharomyces yeasts—Hanseniaspora uvarum grows rapidly and resists inhibition by Saccharomyces cerevisiae, and can even slow it. Together with Conacher’s synthetic consortia and Sizzano’s real-world fermentations, the studies portray fermentation as a dynamic, multispecies ecosystem and illustrate cytometry’s versatility from enumeration to phenotyping.

Among functional assays, the basophil activation test (BAT) is increasingly central to the diagnosis of IgE-mediated allergies. Groffmann et al. (contribution 11) advance the test using an automated bin-based analysis of wheat allergy: in children, allergic subjects exhibited higher activation, with the BAT reaching moderate performance (AUC 0.71–0.73) generally exceeding extract-based specific IgE, though Tri a 19-specific IgE remained the best option (AUC 0.97); in adults, the test did not discriminate. CD32 downregulation emerges as promising in vivo readout.

Reliable interpretation depends on the recognition of pitfalls: the review by Ebo et al. [30] assesses the non-releaser phenotype—the 10–20% whose basophils fail to respond—distinguishing intrinsic causes (Syk deficiency, defective IgE signaling) from analytical ones and proposing standardized nomenclature for reporting inconclusive results, underscoring that an accurate BAT depends as much on methodological rigor as on technology.

Payne et al. [31] extend the assay to alcohol hypersensitivity in aspirin-exacerbated respiratory disease and chronic rhinosinusitis, showing that specific polyphenols—not ethanol itself—activate basophils and eosinophils. By implicating molecules not traditionally regarded as allergens, this reframes the BAT as an integrated platform for probing immune reactivity across food and respiratory disease.

Finally, Mzoughi et al. [32] illustrate both the promise and limits of the BAT in drug hypersensitivity: in a real-world Tunisian cohort, it offered a feasible alternative to skin testing and drug-specific IgE for antibiotics and non-steroidal anti-inflammatory drugs; the performance was modest (9.4% sensitivity). The absence of uniform protocols reinforces the cluster’s recurring message: functional basophil testing will reach the clinic only with rigorous standardization methodologies.

As immunophenotyping relies on high-dimensional panels and cryopreserved samples, antibody clone selection becomes decisive. Serra et al. (contribution 7) address this with FlowCLOc, a data-driven antibody selection resource: comparing 438 clones across fresh and cryopreserved blood and peripheral blood mononuclear cells (PBMCs), they demonstrate that freezing affects the detection of over half the markers by >20%, rendering some clones (e.g., CD19 HIB19, CD3 HIT3α) unreliable. Its interactive catalog allows researchers to pre-select robust antibodies, improving reproducibility.

FlowCLOc complements advances in panel design: the standardized 17-color spectral workflow of Kiel et al. [6] optimizes resolution once markers are chosen, while the clinical COMIP-001 13-antibody, 12-color panel [33] reveals how rigorous clone selection underpins diagnostic robustness and inter-operator reproducibility. FlowCLOc adds a missing pre-analytical layer: before optimizing fluorochromes, one must confirm that each clone performs in the intended sample matrix and cell subset.

Together, these sketch a roadmap for high-dimensional, cryopreservation-based immunophenotyping: empirically grounded antibody selection (FlowCLOc), spectral optimization and quality control (Kiel et al. [6]), and validated clinical panels (COMIP-001). Extensions of FlowCLOc to intracellular and activation-induced epitopes and linkage to diagnostic performance would further bridge research-grade profiling and translatable clinical assays.

Quantitative flow cytometry (QFCM) has the potential to transform immunophenotyping into a metrological discipline, yet Poncelet et al. (contribution 6) note that it remains underused. They set out the foundations of reliable measurements—MESF/ABC-bead calibration, saturated no-wash staining, stable reagents, harmonized instrumentation—and demonstrate that major platelet glycoproteins (GPIIb/IIIa, GPIb/IX/V, GPVI, P-selectin) are stable enough to serve as blood-borne standards for diagnosing inherited platelet disorders.

Building on this, Spurgeon [34] reframes platelet flow cytometry as a multidimensional clinical platform rather than a receptor-counting assay: modern workflows characterize platelet subpopulations, assess complement deposition and immune complex binding, and evaluate function across immune-mediated and thrombotic disorders—with reliable antigen density measurements as the prerequisite for interpreting pathological shifts.

A companion perspective [35] demonstrates that flow cytometry enables targeted platelet function testing in whole blood with minimal volumes—valuable in pediatrics, thrombocytopenia, and resource-limited settings—where combining activation markers, signaling readouts, and microvesicle release with quantitative antigen density distinguishes low receptor density from signaling defects.

Looking ahead, Yao et al. [7] highlight platelet mitochondria as an emerging therapeutic target, proposing mitochondria-targeted antioxidants as antiplatelet strategies and platelet-derived mtDNA as a thrombosis biomarker; QFCM-based readouts (membrane potential, ROS, mitochondrial protein expression) could serve as pharmacodynamic markers in early-phase trials.

Together these trace a trajectory from counting to context and from diagnosis to therapy monitoring, with absolute antigen densities anchoring interpretation. Realizing this objective requires cross-laboratory standardization, matched calibrators, and validated reference ranges—this convergence marks 2026 as a plausible turning point for platelet QFCM.

Macrophages are a further theme, anchored by the review by Falda (contribution 10), which frames macrophage plasticity as a functional continuum shaped by origins and the microenvironment rather than a binary M1/M2 dichotomy. It maps regulators of macrophage identity—TLR and cytokine signaling, microRNA networks, metabolic rewiring, and histone lactylation—across cancer, autoimmunity, infection, and metabolic and neurological disease, arguing that standardized high-dimensional panels enable biomarker-driven stratification.

Several papers then apply this framework. Wan et al. [36] examine the “metabolism–immunity–bone” axis—linking macrophage metabolic reprogramming (glucose, lipid, amino acid pathways; HIF-1α–glycolysis signaling) to dysregulated bone remodeling in osteoporosis, rheumatoid arthritis, and osteoarthritis—and propose metabolic modulators and targeted nanomedicine to steer macrophages toward bone regeneration.

Wang et al. [8] review molecular positron emission tomography (PET) imaging of tumor-associated macrophages (TAMs). CSF1R, CD206, CD163, and CCR2 tracers—with nanobody- or peptide-based probes—enable whole-body, high-specificity quantification and phenotyping of macrophage subsets, overcoming the limits of biopsy and non-specific [^18^F]FDG signals and positioning the use of TAM-PET for patient stratification, therapy monitoring, and theranostics.

Li et al. [37] consolidate the therapeutic landscape into a multilayered anti-TAM framework: blockades of monocyte recruitment (CCL2–CCR2, CSF-1/CSF-1R, CXCL12–CXCR4); direct depletion (CSF-1R inhibitors, clodronate liposomes, trabectedin); phenotypic reprogramming (TLR and CD40 agonists, PI3Kγ and STAT3 inhibitors); CAR-macrophages; and nanotechnology-based delivery. These synergistically perform with anti-PD-1/PD-L1, anti-VEGF, radiotherapy, and chemotherapy, enhancing antigen presentation and T-cell activation.

Complementing this, Li et al. [9] contribute a subset-centric, temporally resolved perspective grounded in single-cell transcriptomics, advancing precision approaches—pathway-specific targeting, adoptive transfer of reparative subsets, and engineered CAR-M—that move the field from phenotypic taxonomy to data-driven intervention.

Finally, Popov et al. [38] demonstrate how biomaterials affect macrophage behavior: surface chemistry, ECM motifs (e.g., RGD), stiffness, porosity, and degradation kinetics tune adhesion and polarization, while nanoparticles, hydrogels, and scaffolds enable active reprogramming via stimulus-responsive delivery across chronic wounds, tumors, atherosclerosis, and diabetic tissue.

## Figures and Tables

**Figure 1:**
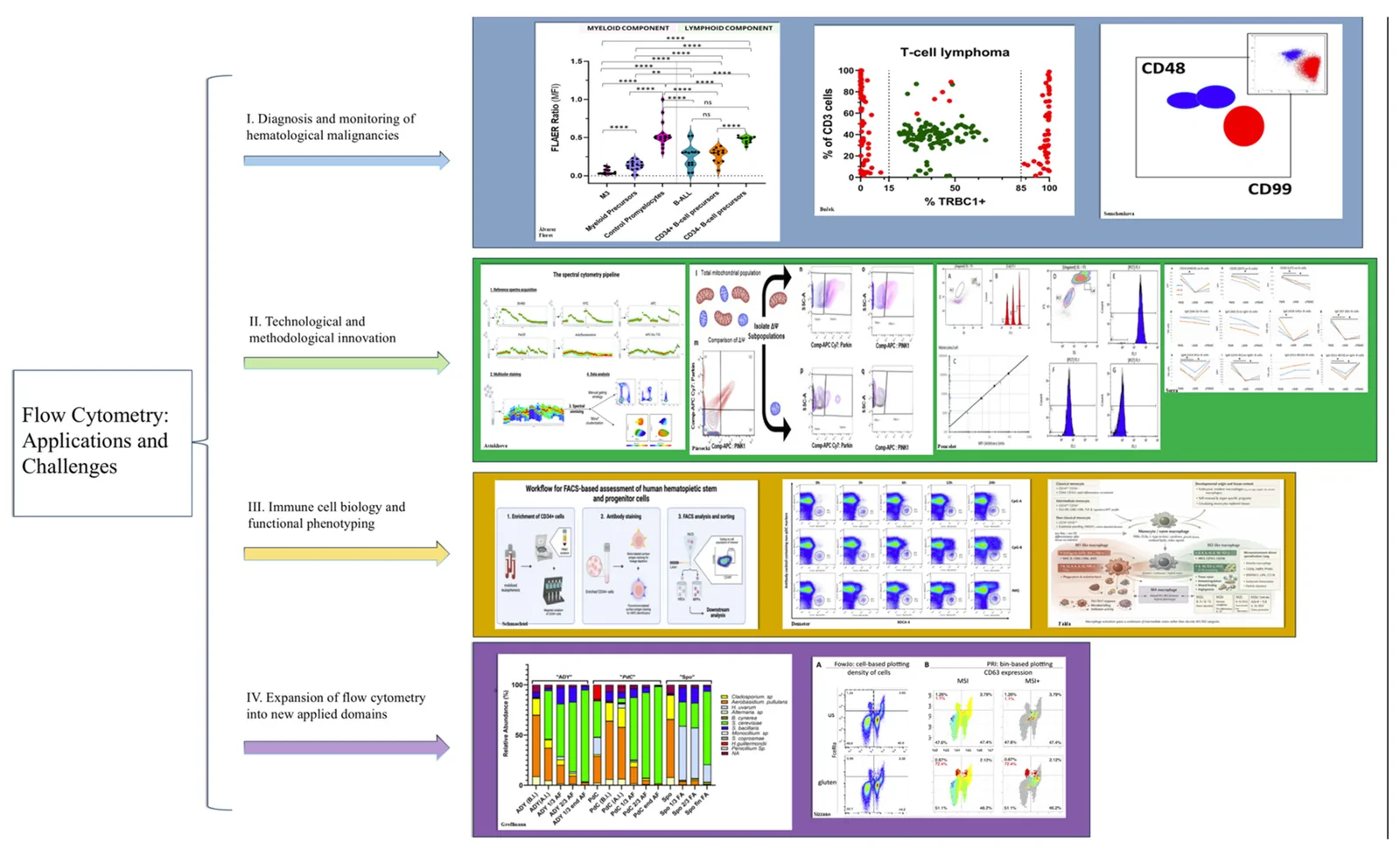
Visual synthesis of the Special Issue “Flow Cytometry: Applications and Challenges.” The twelve contributions are organized into thematic quadrants—diagnosis and monitoring of hematological malignancies, technological and methodological innovation, immune cell biology and functional phenotyping, and expansion into new applied domains. Within each quadrant, the panel for each contribution pairs its key application with the current challenge it identifies, echoing the dual focus of the collection.
